# Proceeding From *in vivo* Functions of Pheromone Receptors: Peripheral-Coding Perception of Pheromones From Three Closely Related Species, *Helicoverpa armigera, H. assulta*, and *Heliothis virescens*

**DOI:** 10.3389/fphys.2018.01188

**Published:** 2018-08-30

**Authors:** Bing Wang, Yang Liu, Gui-Rong Wang

**Affiliations:** State Key Laboratory for Biology of Plant Diseases and Insect Pests, Institute of Plant Protection, Chinese Academy of Agricultural Sciences, Beijing, China

**Keywords:** sex pheromones, *Helicoverpa armigera*, *Helicoverpa assulta*, *Heliothis virescens*, pheromone receptors, transgenic fly

## Abstract

Three closely related species, *Helicoverpa armigera*, *H. assulta*, and *Heliothis virescens* from Lepidoptera Noctuidae, are used as a model system for exploring sexual communication and species isolation. Pheromone receptors (PRs) previously discovered in model moth species include seven in *H. armigera*, six in *H. assulta*, and six in *H. virescens*. PRs named OR6, OR13, and OR16 among these species were found to be functional, characterized by an *in vitro Xenopus* oocytes system. Using an *in vivo* transgenic fly system, functional assays of OR6, OR13, and OR16 clades from three closely related Noctuidae species showed that OR13 function was highly conserved, whereas OR6 and OR16 exhibited functional divergence. Similar results were produced from assays in the *Xenopus* oocytes system. Combined with earlier behavioral results and electrophysiological recordings, we found corresponding relationships among pheromones, PRs, and neurons at the periphery sensory system of each species. Our results provide vital information at the neuronal and molecular level, shedding insight into the sexual communication of closely related species in Lepidoptera.

## Introduction

Moth sex pheromones are released by female moths to attract conspecific males, allowing for long-distance mating attraction. Reception of moth sex pheromones among closely related species is complicated by diverse releasing and receiving pheromone signals, as well as varying components, quantities, and ratios of moth sex pheromones (Berg et al., 1995; Hansson et al., 1995; Baker et al., 2004; Wang et al., 2010; Vasquez et al., 2011; Zhang and Löfstedt, 2015). Sexual communication of closely related moth species in Lepidoptera Noctuidae has been studied over a few decades as a model system for exploring sex pheromone recognition and species isolation mechanisms (Kehat and Dunkelblum, 1990; Almaas and Mustaparta, 1991; Berg et al., 1998; Baker et al., 2004; Krieger et al., 2004; Groot et al., 2006; Gould et al., 2010; Wang et al., 2010; Wu et al., 2015; Chang et al., 2016). However, there is not sufficient evidence to explain how intra- and interspecific sexual communication signals of closely related species are discriminated (Vasquez et al., 2011; Zhang et al., 2016).

Three Lepidoptera species across two genera, *Helicoverpa armigera* and *H. assulta* in Helicoverpa and *Heliothis virescens* in Heliothis, are phylogenetically closely related and have been thoroughly studied. *H. armigera* and *H. assulta* are sympatrically occurring species found throughout different regions of China, and *Heliothis virescens* is found in America and other countries (Wang et al., 2005; Cho et al., 2008). Sex pheromone blends found in females of these three species overlap in several sex pheromone components. The major component is (*Z*)-11-hexadecenal (*Z*11-16:Ald) in *H. armigera* and *H. virescens*, and (*Z*)-9-hexadecenal (*Z*9-16:Ald) in *H. assulta* (Vetter and Baker, 1983; Cork et al., 1992; Baker et al., 2004), occurring in different ratios with other minor components (Nesbitt et al., 1979; Cork et al., 1992; Chang et al., 2016). Five additional compounds were identified in gland extracts of *H. armigera* females: hexadecanal (16: Ald), hexadecanol (16: OH), (Z)-11-hexadecenol (Z11-16:OH), (Z)-7-hexadecenal (Z7-16:Ald), and (Z)-9-tetradecenal (Z9-14:Ald) (Nesbitt et al., 1979; Dunkelblum et al., 1980; Kehat and Dunkelblum, 1990). Similarly, seven compounds were identified from gland extracts of *H. assulta* females: 16:Ald, (Z)-9-hexadecenyl acetate (Z9-16:OAc), (Z)-11-hexadecenyl acetate (Z11-16:OAc), hexadecanyl acetate (16:OAc), (Z)-9-hexadecenol (Z9-16:OH), Z11-16:OH, and hexadecanol (16:OH) (Cork et al., 1992; Berg and Mustaparta, 1995). However, the *H. virescens* female glands only produce six aldehydes and alcohols rather than acetates, including tetradecanal (14:Ald), Z9-14:Ald, Z7-16:Ald, *Z*9-16:Ald, Z11-16:OH, 16:Ald (Tumlinson et al., 1975; Klun et al., 1980; Vetter and Baker, 1983; Ramaswamy et al., 1985; Teal et al., 1986; Groot et al., 2006, 2009, 2013).

Field tests and behavior experiments have shown that binary pheromone blends of Z11-16:Ald and Z9-16:Ald effectively attract *H. armigera* males (Kehat et al., 1980; Kehat and Dunkelblum, 1990). Z11-16:OH significantly reduced catches but 16: Ald acted in opposite function when mixed with the sex pheromone principal of *H. armigera* (Wu et al., 1997). In addition, the pheromone component Z9-14:Ald (found in *H. armigera* but not *H. assulta*) mixed with binary pheromone blends of Z11-16:Ald and Z9-16:Ald caught more *H. armigera* males at lower concentrations compared to *H. assulta*, whereas it significantly inhibited the attraction behavior of *H. armigera* at higher concentrations (Gothilf et al., 1978; Kehat and Dunkelblum, 1990; Zhang et al., 2012; Wu et al., 2015). In *H. assulta*, addition of Z9-14:Ald or Z9-16:OH to the principal pheromone blend in certain amounts significantly reduced trap catch of male *H. assulta* in both field and laboratory experiments (Cork et al., 1992; Park et al., 1994; Boo et al., 1995). However, when Z9-16:OAc and Z11-16:OAc were added to binary pheromone blends of Z9-16:Ald and Z11-16:Ald at a certain ratio the male *H. assulta* would show attractive and mating behavior (Cork et al., 1992; Park et al., 1994). In *H. virescens*, males use *Z*11-16:Ald and *Z*9-14:Ald as the principal pheromone blend for upwind flight behavior (Vetter and Baker, 1983; Ramaswamy et al., 1985). When 16:Ald was added to pheromone blends of Z11-16:Ald and Z9-14:Ald, close-range sexual behaviors of male moths usually increased (Vetter and Baker, 1983). However, *H. virescens* does not produce acetates compared to *H. armigera* and *H. assulta* (Tumlinson et al., 1975; Klun et al., 1980; Vetter and Baker, 1983; Ramaswamy et al., 1985; Teal et al., 1986; Groot et al., 2006).

In previous studies, electrophysiological responses of sex pheromone have been recorded from a single cell within trichoid sensillum of male antennae in *H. armigera*, *H. assulta*, and *H. virescens*, showing specific neuron responses activated by sex pheromones (Baker et al., 2004; Gould et al., 2010; Wu et al., 2015; Chang et al., 2016; Xu et al., 2016). Genes encoding pheromone receptors (PRs), expressed on the dendritic membrane of specific olfactory receptor neurons (ORNs) in trichoid sensilla of adult male antennae, are vital to the reception of conspecific sex pheromones (Baker, 2009; Wang et al., 2010; Zhang and Löfstedt, 2015). PRs have been identified and characterized by species from genomic databases, cDNA-library screenings, and the antennal transcriptome sequencing, with seven PRs in *H. armigera*, six in *H. assulta*, and six in *H. virescens* (Krieger et al., 2004; Liu et al., 2012; Zhang et al., 2015a). The function and localization of PRs were demonstrated by electrophysiology methods and *in situ* hybridization studies (Krieger et al., 2004, 2009; Grosse-Wilde et al., 2007; Baker, 2009; Wang et al., 2010, 2016; Liu et al., 2013a; Jiang et al., 2014; Chang et al., 2016; Xu et al., 2016).

To date, several strategies for deorphanizing Lepidoptera PRs have been developed both *in vitro* and *in vivo* systems (**Supplementary Table S1**). The most common method to study insect ORs is *in vitro* heterologous expression in *Xenopus* oocytes (de Fouchier et al., 2014; Zhang and Löfstedt, 2015; Cui et al., 2018). Another transgenic fly lines have been used to assay OR function since 2003. The earliest system for studying OR functions was the *Drosophila* “empty neuron” system (Dobritsa et al., 2003). The advantage of this system is that the target OR gene is expressed in the *Drosophila* “empty neuron,” offering an actual cellular environment and allowing heterologous OR coupling with endogenous Orco. At the same time, the odorants can be delivered in gaseous form and combined with the *Drosophila* OBPs, *in vivo* (Hallem et al., 2004; Carey et al., 2010). However, the “empty neuron” system has some limitations for testing other ORs, such as lepidopteran pheromone receptors (Syed et al., 2010). These limitations likely arise due to some essential factors, for instance, sensory neuron membrane proteins (important for pheromone-evoked neuronal activity) are lacking in the ab3A neuron (Benton et al., 2007). However, some studies have proven that the *Or67d*^GAL4^ knock-in system is better for detecting the function of moth pheromone receptors in terms of structural, biochemical, and/or biophysical features of the at1 trichoid sensilla (Syed et al., 2010; Vasquez et al., 2013; Wang et al., 2016).

In this study, we constructed a phylogenetic tree from seven identified Lepidopteran species, and revealed orthology with closely related Noctuidae PRs. According to their evolutionary relationships and functions, we selected three sets of homologous genes, *OR6*, *OR13*, and *OR16*, from *H. armigera*, *H. assulta*, and *H. virescens*, respectively, and predicted highly conserved sequences motifs. Then, we constructed nine transgenic fly lines using the *Or67d*^GAL4^ knock-in system for further functional characterization. Specifically, we compare PR functions between the *Xenopus* oocytes system and the *Or67d*^GAL4^ knock-in system, as well as the relationships between PRs and neurons in the peripheral nervous system. Our results summarize the correlations among pheromones, pheromone receptors, and neurons at the periphery of the sensory system from three closely related species in Lepidoptera, as well as provide information to further detect evolutionary relationships of sex pheromones.

## Materials and Methods

### Insect Rearing

*Drosophila* stocks were fed cornmeal-agar-molasses medium and maintained under a 12 h light: 12 h dark cycle at 25°C and 60% relative humidity. The medium was changed after 10 days. Three to ten-days adults were used to test.

### Fly Strains

Transgenic lines were generated according to standard procedures as described below. The open reading frame encoding OR6/OR13/OR16 genes was cloned into the pVALIUM20 vector (Ni et al., 2011). Independent homozygous UAS-OR lines (with transgene insertions into chromosome II) were generated at the Tsinghua Fly Center (Beijing, China). Driver mutant allele *Or67d^GAL4^* stock was provided by Dr. Barry J. Dickson (Kurtovic et al., 2007). The balancer *w-/w-; sp/CyO; TM3/TM6B* was used to cross with homozygous driver lines. The driver line in the *Or67d^GAL4^* mutant background was then crossed with the UAS-OR balancer line to establish a final homozygous stock *w+/w+; UAS-OR/UAS-OR; Or67d*^GAL4^*/ Or67d*^GAL4^ which expressed OR6/OR13/OR16 genes in at1 sensilla neurons. Each OR6/OR13/OR16 insertion was confirmed by sequencing genomic DNA prepared from mutant lines. The final stock was used for electrophysiological experiments.

### Sequence Analysis and Phylogenetic Tree Construction

The amino acid sequences of OR6, OR13, and OR16 from *H. armigera*, *H. assulta*, and *H. virescens*, respectively, were aligned using ClustalX software (Version 2.1, European Bioinformatics Institute). Dendrograms were labeled by FigTree software^1^. The transmembrane domains of PR6, PR13, and PR16 were predicted using TMHMM Server Version 2.0^2^. The phylogenetic tree of PRs genes in different Lepidoptera species was constructed by RaxML version 8 with Jones-Taylor-Thornton amino acid substitution model (JTT) (Stamatakis, 2014). Node support was assessed using a bootstrap method based on 1000 replicates. The PR and Odorant receptor co-receptor (Orco) data set contained 38 PR and seven Orco sequences identified in Lepidoptera [eight from *H. armigera* (Liu et al., 2012), seven from *H. assulta* (Zhang et al., 2015a), seven from *H. virescens* (Wang et al., 2010), eight from *B. mori* (Nakagawa et al., 2005; Wanner et al., 2007), five from *S. exigua* (Liu et al., 2013a), five from *S. litura* (Zhang et al., 2015b), and five from *S. littoralis* (Montagné et al., 2012; de Fouchier et al., 2015)]. The phylogeny of the seven moth species above was constructed on the basis of cytochrome oxidase subunit I (COI) genes.

### Motif-Pattern Analysis

The motif-pattern analysis of proteins was performed broadly using the MEME online server (MEME Suite Version 4.11.2)^3^. A total of nine PRs from *H. armigera*, *H. assulta*, and *H. virescens* were selected to predict the conserved motif pattern. The parameter settings were as follow: maximum number of motifs was eight, minimum motif width was six, maximum motif width was 15, and Expectation maximization (EM) improvement threshold was 10^-5^.

### Single Sensillum Recordings

Using a transgenic *in vivo* system, the OR6, OR13, and OR16 genes across three Heliothis/Helicoverpa species were respectively expressed in at1 neurons of *Drosophila*, and the resulting *UAS-OR* flies were crossed with a mutant knock-in allele *Or67d*^GAL4^ driver line. Extracellular electrophysiological recordings were performed on single at1 sensilla of one to 10 day old flies. The antenna was fixed using standard procedures (de Bruyne et al., 2001; Syed et al., 2006). The reference electrode was placed in the fly eye, under a microscope (LEICA Z16 APO, Germany) at 920 × magnification. Action potentials were recorded by inserting a tungsten wire electrode in the base or in the shaft of a sensillum of the fly antenna. Signals were amplified 10× by a high impedance pre-amplifier (IDAC-4 USB System, Syntech, Kirchzarten, Germany), sent to a PC via an analog-digital converter, and analyzed off-line with AUTOSPIKE v. 3.9 software (Syntech, Kirchzarten, Germany). The filter was set with a 500 Hz low cutoff and a three kHz high cutoff. AC signals were recorded for 10 s, starting 1 s before stimulation. Responses were calculated by counting the number of action potentials 1 s after stimulation (with a delay of 200 ms to allow the odorant to travel down the airstream), and subtracting the number counted in the second before stimulation. Three dimensional bar charts were created in SigmaPlot Version 12.5 (SYSTAT, San Jose, CA, United States). Heatmaps of different PR functions activated by sex pheromone components and analog were generated by Heml 1.0 software (Deng et al., 2014).

### Odor Stimulation

In total, nine sex pheromone components and analogs, Z9-14:OAc, Z9-16:OAc, Z11-16:OAc, Z9-14:Ald, Z9-16:Ald, Z11-16:Ald, Z9-14:OH, Z9-16:OH, and Z11-16:OH, were used to screen *in vivo* functions of all three types ORs across three Heliothis/Helicoverpa species with paraffin oil as a control. Aliquots of sex pheromone components were dissolved in paraffin oil (v/v), and 10 μL of each solution were loaded onto a 5 × 40 mm Whatman filter paper strip, which was placed inside a Pasteur pipette. Paraffin oil alone was tested as a negative control. For dose-response relationships, serial dilutions were made in increasing doses of 0.001, 0.01, 0.1, 1, 10, and 100 μg/μL and loaded on separate filter paper strips. Each preparation was held in a humidified continuous air flow delivered by the Syntech Stimulus controller (CS-55 model, Syntech) at 1.4 L/min. Stimulus pulses were added for 300 ms. During stimulation, the compensatory flow was switched off.

## Results and Discussion

### Phylogenetic Analysis Reveals Orthology With Closely Related Noctuidae PRs

**FIGURE 1:**
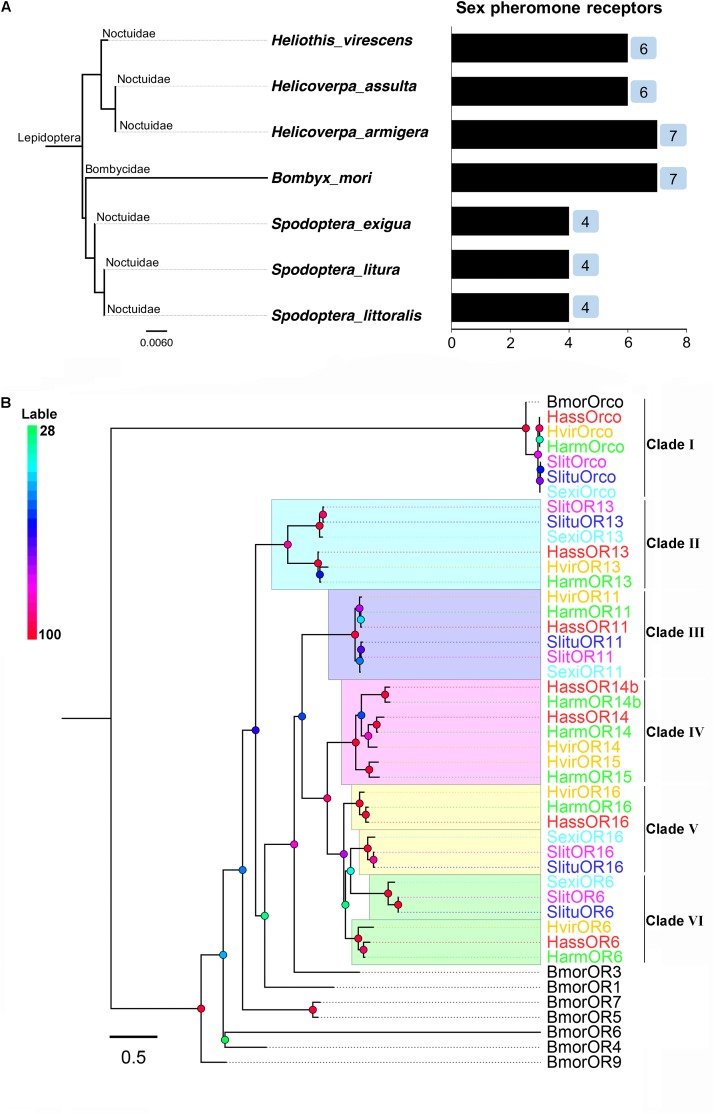
The phylogeny of pheromone receptors (PRs) from seven moth species. **(A)** Phylogeny and number of PRs identified by species, including *Helicoverpa armigera*, *H. assulta*, *Heliothis virescens*, *Bombyx mori*, *Spodoptera exigua*, *S. litura* and *S. littoralis.* **(B)** A phylogenetic tree of PR genes in different Lepidoptera species. Six clades (I to VI) are shown in this tree representing Orco, OR13, OR11, OR14/15, OR16, and OR6 clades, respectively.

In some Lepidoptera species (especially in the superfamily Noctuidae), the number of PRs revealed, identified, and characterized by species were four in *Spodoptera exigua* (OR6, 11, 13, 16), four in *Spodoptera litura* (OR6, 11, 13, 16), four in *Spodoptera littoralis* (OR6, 11, 13, 16), seven in *H. armigera* (OR6, 11, 13, 14, 14b, 15, 16), six PRs in *H. assulta* (OR6, 11, 13, 14, 14b, 16), and six in *H. virescens* (OR6, 11, 13, 14, 15, 16). In addition to Noctuidae species, seven PRs (OR1, 3, 4, 5, 6, 7, 9) were identified and characterized in *Bombyx mori*, belonging to Bombycidae (Nakagawa et al., 2005; Wanner et al., 2007; Wang et al., 2010, 2016; Liu et al., 2012; Montagné et al., 2012; Liu et al., 2013a,b; Jiang et al., 2014; de Fouchier et al., 2015; Zhang et al., 2015a,b; Chang et al., 2016) (**Figure 1A** and **Supplementary Table S1**).

**FIGURE 2:**
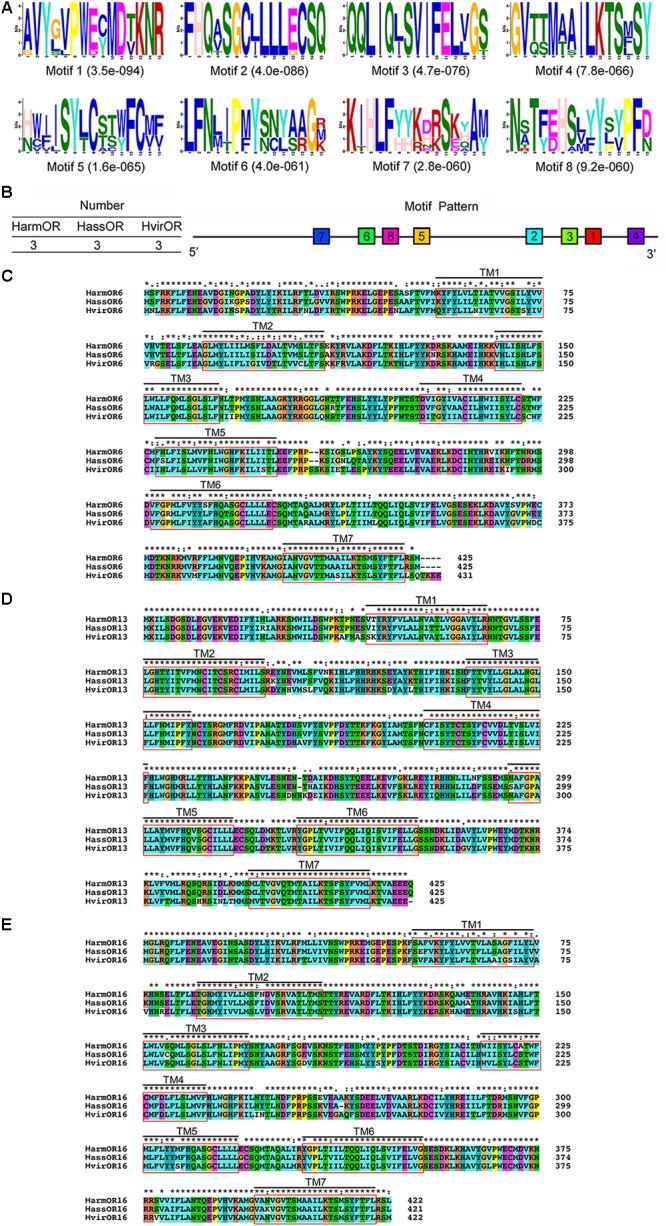
Motif analysis of pheromone receptors (PRs) identified from three closely related Lepidoptera species, and the alignment of amino acid sequence of three set of PRs. **(A)** The eight motif-pattern discovered in nine PRs from *Helicoverpa armigera*, *H. assulta*, and *Heliothis virescens*. **(B)** The locations of each motif-pattern on the predicted protein sequence from N-terminal to C-terminal. Smaller numbers indicate higher conservation. **(C)** The alignment of amino acid sequence of clade OR6 from *H. armigera*, *H. assulta*, and *H. virescens*. TM1-TM7 indicates seven transmembrane domains. Harm: *H. armigera*; Hass: *H. assulta*; Hvir: *H. virescens*. **(D)** The alignment of amino acid sequence of clade OR13 from *H. armigera*, *H. assulta*, and *H. virescens*. **(E)** The alignment of amino acid sequence of clade OR16 from *H. armigera*, *H. assulta*, and *H. virescens*.

Full-length amino acid sequences of candidate PRs genes were used to construct a phylogenetic tree from seven identified lepidopteran species including *B. mori*, *H. armigera*, *H. assulta*, *H. virescens*, *S. exigua*, *S. litura*, and *S. littoralis* (**Figure 1B**). Orthologous genes of the highly conserved co-receptor Orco, were clustered together as Clade I. As expected, sequence identity among them was very high. Another five orthologous clades were shown as noctuids species in Clade II-VI, representing clades OR6, OR11, OR13, OR14/14b/15, and OR16 (**Figure 1B**). The amino acid sequences of PRs across various noctuids species in OR13 clade are quite conserved, showing functional conservation; the sequences of OR6 or OR16 clade are relatively less conserved, exhibiting functional differentiation (**Figure 2**) (Wang et al., 2010; Liu et al., 2013b; Jiang et al., 2014; de Fouchier et al., 2015).

### Three Sets of Homologous PR Genes Selected and Cloned From Closely Related Species

Evolutionarily, *H. armigera, H. assulta*, and *H. virescens* are highly related compared with other Lepidopteran species (Wang et al., 2005; Cho et al., 2008). PRs of these three species could respond to overlapping sex pheromone components (Wang et al., 2010; Liu et al., 2013b; Jiang et al., 2014). Thus, studying evolutionary relationships among PRs in *H. armigera* and related species will provide valuable information on reproductive isolation.

Based on previous studies of PRs across Heliothis/Helicoverpa species, several pheromone components were used to determine response profiles of all PRs across Heliothis/Helicoverpa species, mainly using an *in vitro* two-electrode voltage-clamp system (Wang et al., 2010, 2016; Liu et al., 2013b; Jiang et al., 2014; Chang et al., 2016; Xu et al., 2016). We found that none of the OR11 and OR15 PRs across three Heliothis/Helicoverpa species were activated by any pheromone component tested. However, only HvirOR14 of all OR14 PRs across three Heliothis/Helicoverpa species showed response, and was activated by Z11-16:OAc and Z9-14:Ald. Similarly, OR14b from *H. virescens* was not identified (**Supplementary Table S2**). Therefore, we selected homologous OR6, OR13, and OR16, which play an important role in mating, for comparing the functions across three species. Three sets of homologous PR genes (total of nine PRs) were cloned from cDNA sequences according to the genomic database and antennal transcriptome sequence (Krieger et al., 2004; Liu et al., 2012; Zhang et al., 2015a). Subsequently, all genes were subcloned into the expression vector of the transgenic fly for further functional screening.

### Sequence Analysis of Noctuidae PRs Genes

According to amino acid sequences of orthologous PR genes in the closely related species, *H. armigera, H. assulta*, and *H. virescens*, three multiple sequence alignments (OR6, OR13, and OR16) revealed relatively conserved characteristics among orthologous PRs. Each alignment contained seven transmembrane domains (**Figures 2C–E**), with sequence identities of 89.95, 95.54, and 94.08% corresponding to OR6, OR13, and OR16 alignments, respectively.

Nine PR sequences were used to predict highly conserved motifs. A total of eight motifs composed the most common pattern of sequence “7-6-8-5-2-3-1-4,” which represented traits with three types of ORs in *H. armigera, H. assulta*, and *H. virescens*, respectively (**Figure 2B**). The most typical conserved sequence patterns were located in the conserved C-terminal region as (A/G)-V-Y-(G/L/S)-(V/L)-P-W-(E/D)-(C/Y)-M-D-(T/V)-K-N-R in motif 1, F-H-Q-(A/Y/T)-S-G-C-(L/I)-L-L-L-(E/G)-C-S-Q in motif 2, Q-Q-L-I-Q-(L/I)-S-V-I-F-E-L-(V/L)-G-(S/T) in motif 3, and G-V-(T/Q)-(T/S)-M-(A/T)-(A/S)-I-L-K-T-S-(M/E)-S-Y in motif 4 (**Figure 2A**). The functions of these motifs were thought to be important in protein-protein interactions (Miller and Tu, 2008), especially in the formation of the OR/Orco heteromeric complex (Benton et al., 2006; Vasquez et al., 2013). In addition, another four motifs, motif 5 (H/N)-(W/C/V)-(I/F/V)-(I/L)-S-Y-(L/T)-C-(S/T/A)-(T/S/C)-(W/Y)-F-C-(M/Y)-(F/Y), motif 6 L-F-N-(L/M/I)-(I/T)-P-(M/F)-Y-(S/N)-(N/C)-(Y/L)-(A/S) -(A/R)-G-(R/M/K), motif 7 K-(I/T)-H-L-F-(Y/H)-(Y/H)-(K/R)-(D/H/N/E)-(R/K)-S-(K/E/D)-(Y/H/Q/A)-A-(M/Y), and motif 8 N-(S/A/T/R)-T-(F/Y)-(E/D)-H-(S/A)-(L/V/M)-(Y/F)-Y-(S/L/P)-(Y/V)-P-F-(D/N), had lower conservation and exhibited more sequence variation. It is possible that some amino acid residues were highly variable, resulting in functional differentiation. However, the reason for evolutionary differences of PRs presented in closely related species remains unclear.

### *In vivo* Functional Assays of Closely Related Noctuidae PRs

**FIGURE 3:**
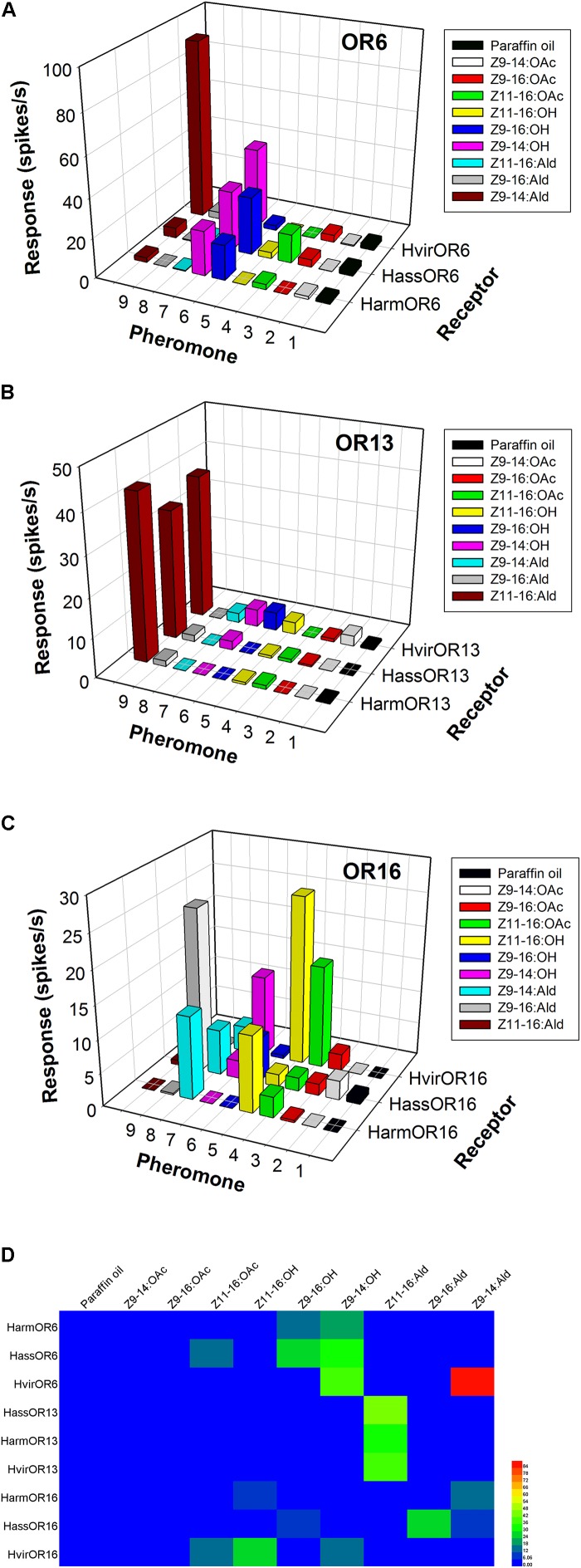
Odor coding of pheromone receptors (PRs) from three closely related species. **(A)** Responses of OR6-expressing neurons in at1 sensilla of transgenic flies. **(B)** Responses of OR13-expressing neurons in at1 sensilla of transgenic flies. **(C)** Responses of OR16-expressing neurons in at1 sensilla of transgenic flies. **(D)** Heatmap of response spectra of PR-expressing neurons in at1 sensilla of transgenic flies.

**FIGURE 4:**
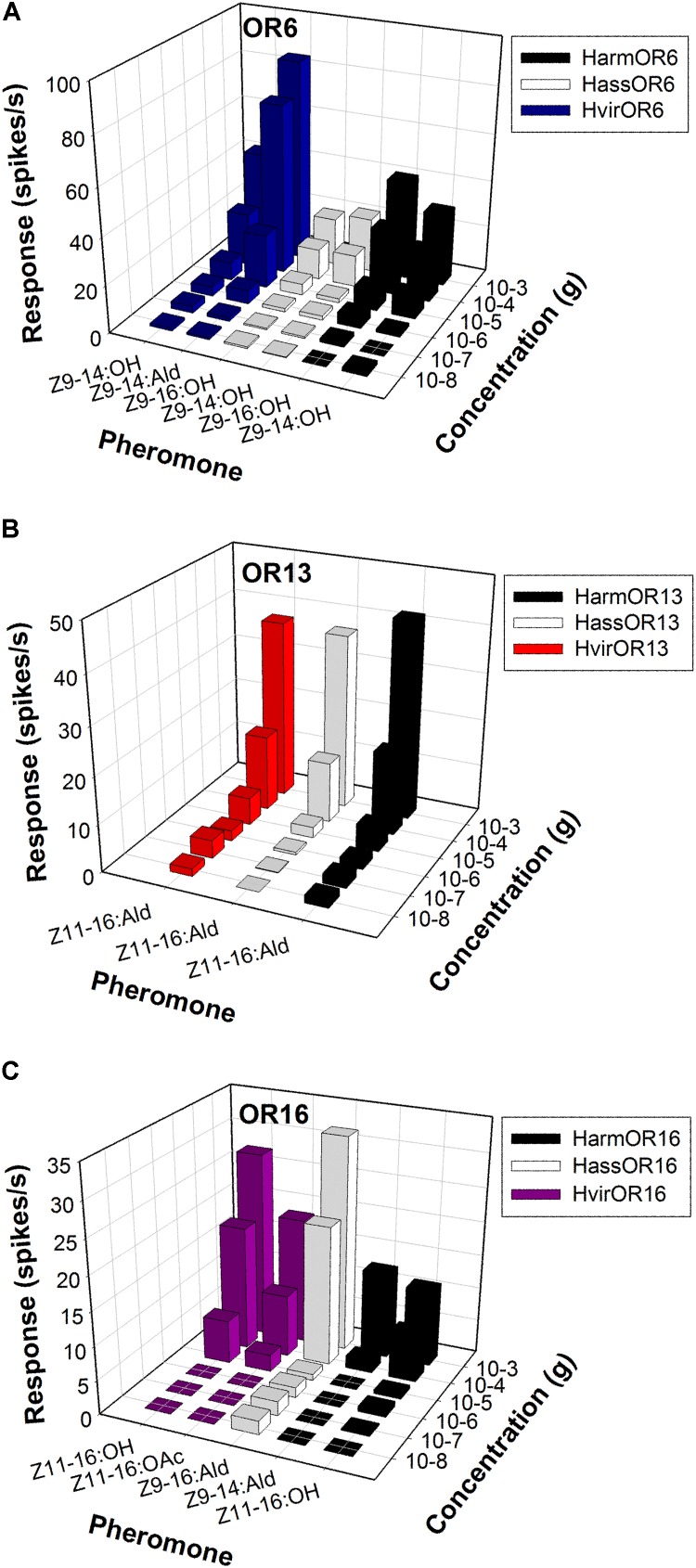
Odor coding of pheromone receptors from three closely related species across concentrations. **(A)** Dose-responses of OR6-expressing neurons in at1 sensilla of transgenic flies. **(B)** Dose-responses of OR13-expressing neurons in at1 sensilla of transgenic flies. **(C)** Dose-responses of OR16-expressing neurons in at1 sensilla of transgenic flies.

In *H. armigera* and *H. assulta*, the OR6-expressing neurons in at1 sensilla mainly responded to the sex pheromone component analogs Z9-14:OH and Z9-16:OH, at a dose of 1 mg loaded in the stimulus cartridge, whereas HvirOR6-expressing neurons responded to Z9-14:Ald and analog Z9-14:OH (**Figures 3A,D** and **Supplementary Figure S1**). In a dose–response experiment, neurons in at1 sensilla started firing at doses as low as 10 ng, with Z9-14:OH and Z9-16:OH EC_50_ values of 3.85 × 10^-5^ and 5.84 × 10^-5^ g in *H. armigera*, 9.66 × 10^-5^ and 5.99 × 10^-5^ g in *H. assulta*, and Z9-14:Ald and Z9-14:OH EC_50_ values of 2.75 × 10^-5^ and 1.26 × 10^-4^ g in *H. virescens* (**Figure 4A** and **Supplementary Figure S1**).

The function of the OR13 gene was highly conserved (**Figure 3D** and **Supplementary Figure S2**). We found that OR13-expressing neurons in at1 sensilla responded specifically to the sex pheromone component Z11-16:Ald at a dose of 1 mg across three Heliothis/Helicoverpa species (**Figure 3B**). Dose–response results showed neurons in at1 sensilla started to respond to Z11-16:Ald at a threshold of 10 ng, and continued to receive stimulation in a concentration gradient up to 1 mg (**Figure 4B** and **Supplementary Figure S2**). The EC_50_ values of Z11-16:Ald were 2.13 × 10^-4^, 2.42 × 10^-4^, and 2.16 × 10^-4^ g in *H. armigera*, *H. assulta*, and *H. virescens*, respectively.

By comparison, the OR16 gene exhibited functional divergence (**Figure 3D** and **Supplementary Figure S3**). In *H. armigera*, the HarmOR16-expressing neurons in at1 sensilla responded to the sex pheromone components Z9-14:Ald, Z11-16:OH, and Z11-16:OAc (**Figure 3C** and **Supplementary Figure S3**). In a dose–response experiment, neurons in at1 sensilla started firing at doses as low as 10 ng, with a Z9-14:Ald EC_50_ value of 1.26 × 10^-3^ g and a Z11-16:OH EC_50_ value of 7.94 × 10^-5^ g (**Figure 4C** and **Supplementary Figure S3**). In *H. assulta*, the HassOR16-expressing neurons in at1 sensilla responded to the sex pheromone components Z9-16:Ald, Z9-14:Ald, and Z9-16:OH (**Figure 3C** and **Supplementary Figure S3**). In addition, neurons in at1 sensilla showed a dose–response, with a Z9-16:Ald EC_50_ value of 8.56 × 10^-5^ g (**Figure 4C** and **Supplementary Figure S3**). In *H. virescens*, the sex pheromone components Z11-16:OH, Z9-16:Ald, Z11-16:OAc, and Z9-14:OH activated the HvirOR16-expressing neurons in at1 sensilla (**Figure 3C** and **Supplementary Figure S3**). The dose–response experiment showed Z11-16:OH and Z11-16:OAc EC_50_ values of 3.89 × 10^-5^ and 8.88 × 10^-5^ g (**Figure 4C** and **Supplementary Figure S3**), respectively.

### PR Functional Comparison Test Between *Xenopus* Oocytes and *Or67d*^GAL4^ Knock-In Systems

According to the previous functional identifications of PRs using the *Xenopus* oocytes system (Wang et al., 2010; Liu et al., 2013b; Jiang et al., 2014; Chang et al., 2016), we summarized the sex pheromone response profiles of PRs across *H. armigera, H. assulta*, and *H. virescens*, and the functions of these PRs using the *Or67d*^GAL4^ knock-in system (**Table 1**). Through a comparative analysis of different methods on functional identification, we found that ligand-binding traits of PRs detected by the *Xenopus* oocytes system are essentially consistent with that of the *Or67d*^GAL4^ knock-in system. This was especially true for functionally conserved PR, OR13, where the best binding-ligand of three orthologous OR13s across Heliothis/Helicoverpa species was the sex pheromone component Z11-16:Ald, regardless of which methods we used. In general, OR/Orco expressed in the *Xenopus* oocytes system was more sensitive to the sex pheromone components. However, the *in vivo Or67d*^GAL4^ knock-in system has generally proven to be more accurate and specific (Wang et al., 2016).

**Table 1:** The comparison of functional characterizations between neurons and odorant receptors.

Species	Sensilla	Type	Neurons	Endogenous System	OR type	*Xenopus* oocyte system	*Or67d*^GAL4^ System
*H. virescens*	Trichoid sensillum	TA	a	Z11-16: Ald^a^	HvirOR13	Z11-16: Ald^b^	**Z11-16: Ald**
						Z9-14: Ald^b^
			b	–	HvirOR11	–^b^	No test
		TB	a	Z9-14: Ald^a^	HvirOR6	Z9-14: Ald^b^	**Z9-14: Ald**
						Z11-16: OH^b^	**Z9-16: Ald^b^**
						Z9-16: Ald^b^	**Z9-14: OH**
			b	–	HvirOR15	–^b^	No test
		TC	a	Z11-16: OAc^a^	HvirOR14	Z11-16: OAc^b^	No test
						Z9-14: Ald^b^
			b	Z11-16: OH^a^	HvirOR16	Z11-16: OH^b^	**Z11-16: OH**
				Z9-14: Ald^a^		Z9-14: Ald^b^	**Z9-16: Ald**
						Z11-16: OAc^b^	**Z11-16: OAc**
							**Z9-14: OH**
*H. armigera*	Trichoid sensillum	TA	a	Z11-16: Ald^c^	HarmOR13	Z11-16: Ald^d^	**Z11-16: Ald**
			b	–	HarmOR11	–^d^	No test
		TB	a	Z9-14: Ald^c^	*HarmOR14b or OR6?*	Z9-14: Ald^c,e,f^	No test
			b	–	HarmOR15	–^d^	No test
		TC	a	Z9-14: Ald^c^	*HarmOR6 or OR14b?*	Z9-16: OH^e^	**Z9-14: OH**
				Z9-16: Ald^c^		Z9-14: Ald^d,e,f^	**Z9-16: OH**
						Z9-16: Ald^d,e,f^	
			b	Z9-14: Ald^c^	HarmOR16	Z11-16: OH^d^	**Z11-16: OH**
				Z11-16: OH^c^		Z9-14: Ald^d^	**Z9-14: Ald**
				Z11-16: OAc^c^			**Z11-16: OAc**
*H. assulta*	Trichoid sensillum	TA	a	Z11-16: Ald^c^	HassOR13	Z11-16: Ald^e^	**Z11-16: Ald**
						Z9-14: Ald^e^	
						Z9-16: Ald^e^	
			b	–	HassOR11	–^e^	No test
		TC	a	Z9-16: Ald^c^	*HassOR6 or OR14b?*	Z9-16: OH^e,f^	**Z9-14: OH**
				Z9-14:Ald^c^		Z9-16: OAc^e^	**Z9-16: OH**
						Z9-14: Ald^e^	
						Z9-16: Ald^e,f^	
			b	Z9-14: Ald^c^	HassOR16	Z9-14: Ald ^e^	**Z9-16: Ald**
				Z9-14: OH^c^		Z11-16: OH ^e^	**Z9-14: Ald**
				Z9-16: OH^c^			**Z9-16: OH**


*“?” Represents uncertainty between neurons and odorant receptors. Italic with underline means the possible alternative. The highlighted with bold font represents the result in this study. Refs: ^*a*^(Baker et al., 2004); ^*b*^(Wang et al., 2010); ^*c*^(Chang et al., 2016); ^*d*^(Liu et al., 2013b); ^*e*^(Jiang et al., 2014); ^*f*^(Yang et al., 2017). It is all predicted the relation between neurons and odorant receptors according to references in this table.*

By comparison, the function of OR6 was relatively divergent. HvirOR6 was mainly tuned to Z9-14:Ald in both *in vivo* and *in vitro* systems. However, HarmOR6/Orco and HassOR6/Orco were all tuned to Z9-14:Ald, Z9-16:Ald, Z9-16:OH, and Z9-14:OH using the *Xenopus* oocytes system, whereas only Z9-16:OH and Z9-14:OH activated HarmOR6/HassOR6 expressing at1 neurons (**Table 1**). These results may be explained by additional factors; for instance, the suitability of ligand concentrations, or whether some OR genes were able to work properly in the *Or67d*^GAL4^ knock-in system. It is pointed out that Z9-14:OH is not a sex pheromone component in any of these closely related species (Nesbitt et al., 1979; Klun et al., 1980;Cork et al., 1992), but instead activates HarmOR6/ HassOR6/ HvirOR6 expressing at1 neurons. This phenomenon requires further investigation.

The function of OR16 was highly divergent and widely tuned to more than three sex pheromone components or analogs, including Z11-16: OH, Z11-16:OAc, and Z9-14:Ald. The major ligands from HarmOR16, HassOR16, and HvirOR16 using both *in vivo* and *in vitro* methods were essentially identical (**Table 1**).

### The Relationship Between PRs and Neurons in the Peripheral Nervous System

Three closely related species use their sensitive olfactory system to specially recognize interspecific-overlapping sex pheromone components. Using previous results from the *Xenopus* oocytes system (Wang et al., 2010, 2016; Liu et al., 2013a; Jiang et al., 2014; Chang et al., 2016) and our results from the *Or67d*^GAL4^ knock-in system combined with *in situ* hybridization and electrophysiological recordings, functional characterization between neurons and odorant receptors were predicted (**Table 1**).

In previous studies, electrophysiological responses of peripheral sex pheromone recognition were recorded from a single sensilla within trichoid sensillum of male antennae in *H. armigera*, *H. assulta*, and *H. virescens* (Baker et al., 2004; Gould et al., 2010; Wu et al., 2015; Chang et al., 2016; Xu et al., 2016). A total of three trichoid sensilla subtypes have been identified to perceive sex pheromone components, A-type, B-type (missing in *H. assulta*) and C-type, each housing two ORNs.

Combined with behavioral results, there may be a correlation between some electrophysiological responses and the functional identification of pheromone receptors. For instance, in *H. armigera*, Z9-14:Ald was previously found to effectively enhance attractions at lower concentrations, and significantly inhibit attraction behavior at higher concentrations (Gothilf et al., 1978; Kehat and Dunkelblum, 1990; Zhang et al., 2012; Wu et al., 2015), whereas Z11-16: OH was found to be a behavioral inhibitor (Wu et al., 1997). Single sensillum recordings showed an “a-spike” ORN (HarmOR6 or HarmOR14b. The predictions of expressed neurons are given for each ORN) in C-type sensillum was tuned to two behavioral agonists, Z9-14:Ald and Z9-16:Ald, while a “b-spike” ORN (HarmOR16) in C-type sensillum was tuned to three behavioral antagonists, Z9-14:Ald, Z11-16:OH, and Z11-16:OAc (Chang et al., 2016, 2017; Yang et al., 2017) (**Table 1**). In *H. assulta*, an “a-spike” ORN (HassOR6 or HassOR14b) in C-type sensillum was tuned to Z9-16:Ald and Z9-14:Ald, while a “b-spike” ORN (HassOR16) in C-type sensillum was tuned to the behavioral antagonist, Z9-14:Ald, and analogs Z9-14:OH and Z9-16:OH (Chang et al., 2016, 2017; Yang et al., 2017). In *H. virescens*, an “a-spike” ORN (HvirOR14) in C-type sensillum was tuned to Z11-16:OAc, while a “b-spike” ORN (HvirOR16) in C-type sensillum was tuned to Z11-16:OH (interspecific inhibitor) and Z9-14:Ald (Almaas and Mustaparta, 1991; Baker et al., 2004; Wang et al., 2010) (**Table 1**).

In general, electrophysiological responses showed an “a-spike” ORN (predicting OR13-expressing neuron) in A-type sensillum across all three species was activated by the sex pheromone component *Z*11-16:Ald, but another “b-spike” ORN (OR11) in A-type sensillum is still uncharacterized (**Table 1**). In A-type sensillum, the functions of expressed ORs are relatively conserved. In addition, the number of A-type sensilla confers a larger proportion of all trichoid sensilla in *H. armigera* than in *H. assulta*, in accordance with the understanding that *Z*11-16: Ald is major sex pheromone component in *H. armigera* (Chang et al., 2016).

One “a-spike” ORN (OR14b or OR6) in B-type trichoid sensillum is known to be mainly tuned to the sex pheromone component Z9-14: Ald, whereas none of ligands activate a “b-spike” ORN (OR15) in B-type sensillum.

Overall, we summarized the relationships among sensilla, neurons, and PRs involving sex pheromone recognition in the peripheral-coding olfactory system of three Heliothis/Helicoverpa species (**Table 1**). It is evident that neuron function in type-A trichoid sensilla completely matched the function of PRs (OR13 and OR11). However, relationships between neurons in type-B or -C trichoid sensilla and PRs did not fully clarified. The *Or67d*^GAL4^ knock-in system used to detect the function of moth pheromone receptor is nearly identical to the *Xenopus* oocytes system (Wang et al., 2016). A few functional differences are observed between PRs and endogenous neurons in moths which may be driven by many factors such as the cell environment, gene expression, lack of accessories, and category and concentration of ligand. In addition, the functions of OR14b or OR6 in *H. armigera* and *H. assulta* still exist differences in previous studies (**Table 1**) (Jiang et al., 2014; Chang et al., 2016; Yang et al., 2017). Therefore, the CRISPR/Cas9 genome editing technique combined with electrophysiological response assays are needed for functional characterization of OR14b (OR6). It is better for elucidation of the molecular and neuronal mechanisms of sex pheromone identification.

### The Evolution of Lepidoptera PRs Selectivity

Three Heliothis/Helicoverpa male species can perceive respective sex pheromone components released from their female pheromone blends. A few hypotheses have been proposed on how variation is generated during pheromone evolution of closely related species, such as the “asymmetric tracking” hypothesis and the gene duplication hypothesis (Phelan, 1992; Gould et al., 2010; Heckel, 2010). However, it is still elusive how subtle variations of sex pheromone components are precisely distinguished by males of different species. Certain moth PRs of closely related species are evolutionarily conserved under strong selective pressure, whereas PRs are more functionally divergent if relaxed from evolutionary constraint (Zhang and Löfstedt, 2013, 2015). The latter is broadly tuned to the behavioral antagonists and agonist, which efficiently increased the specificity and selectivity of interspecific pheromone detection (Zhang and Löfstedt, 2015). This is consistent with our finding that OR16 from three closely related species exhibits largely functional divergences. The function of HarmOR16 from *H. armigera* has been confirmed to be activated by the pheromone antagonist Z11-16:OH, which regulates optimal mating time and influences fecundity (Chang et al., 2017). One study revealed that single mutations in PRs across Asian and European corn borers selectively altered pheromone recognition (Leary et al., 2012). Another study showed that two site mutations of HassOR14b changed ligand selectivity (Yang et al., 2017). Thus, the evolutionary relationship of structure and function of PRs in closely related Lepidoptera species will help reveal the mechanisms underlying reproductive isolation and speciation.

## Author Contributions

BW, YL, and G-RW designed the experiments. BW performed the experiments and analyzed the data. YL and G-RW contributed reagents, materials, and gene identification. BW and G-RW wrote and revised the paper.

## Conflict of Interest Statement

The authors declare that the research was conducted in the absence of any commercial or financial relationships that could be construed as a potential conflict of interest.

## Footnotes

1 http://tree.bio.ed.ac.uk/software/figtree/

2 http://www.cbs.dtu.dk/services/TMHMM/

3 http://meme-suite.org/tools/meme

## Supplementary Material

FIGURE S1 SSR traces from OR6-expressing neurons in at1 sensilla of *Drosophila* in response to pheromone compounds.

FIGURE S2 SSR traces from OR13-expressing neurons in at1 sensilla of *Drosophila* in response to pheromone compounds.

FIGURE S3 SSR traces from OR16-expressing neurons in at1 sensilla of *Drosophila* in response to pheromone compounds.

TABLE S1 Functional characterizations of PR genes in some Lepidopteran species.

TABLE S2 Functional characterizations of PRs in three Heliothis/Helicoverpa species *in vitro.*
