# Supplementary figures and images for: Proceeding From in vivo Functions of Pheromone Receptors: Peripheral-Coding Perception of Pheromones From Three Closely Related Species, Helicoverpa armigera, H. assulta, and Heliothis virescens
Source: Front Physiol. 2018 Aug 30;9:1188. doi: 10.3389/fphys.2018.01188 (PMC6125646; doi:10.3389/fphys.2018.01188)

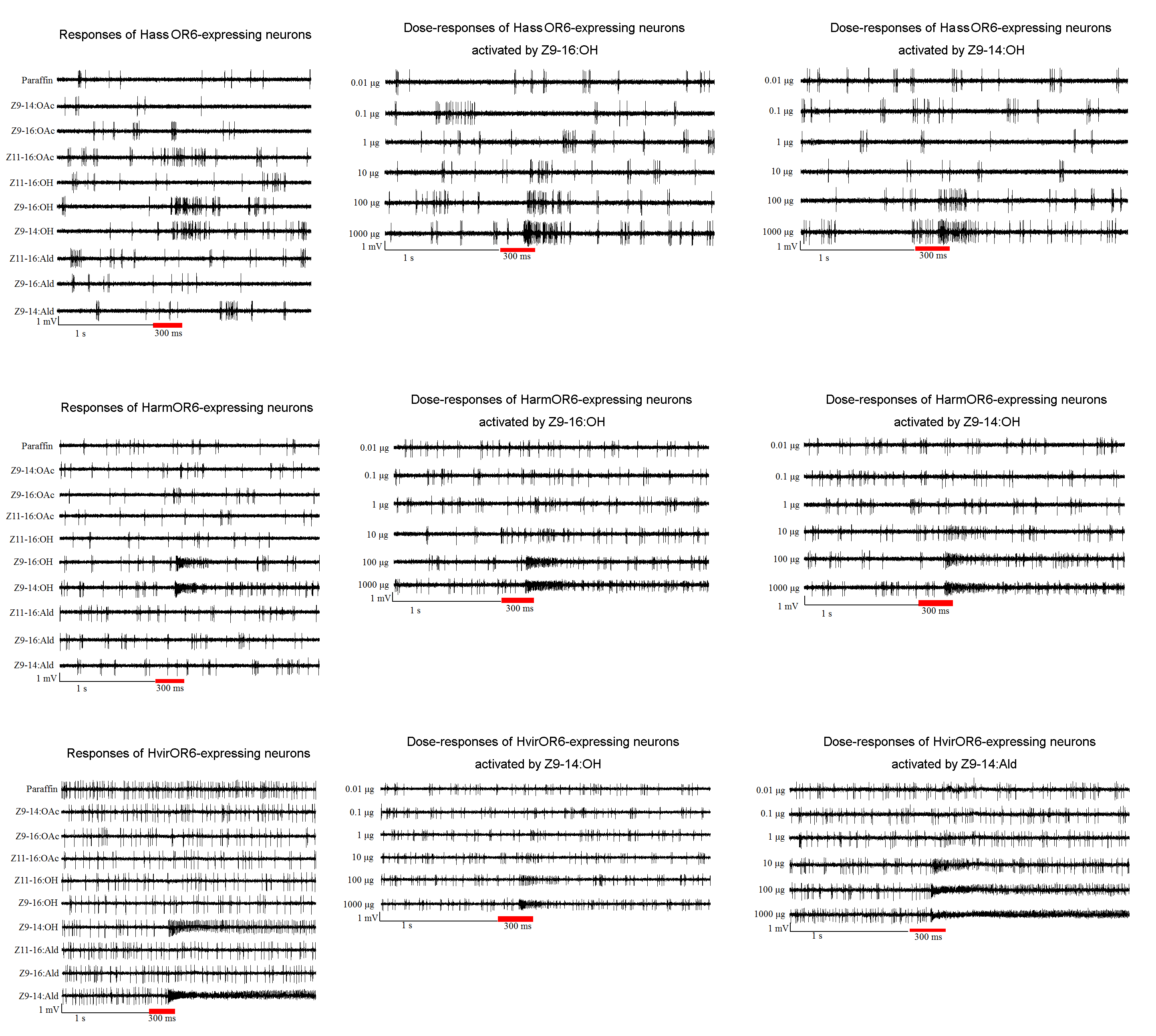

Supplement: FIGURE S1 — SSR traces from OR6-expressing neurons in at1 sensilla of Drosophila in response to pheromone compounds. [file Image_1.TIF]

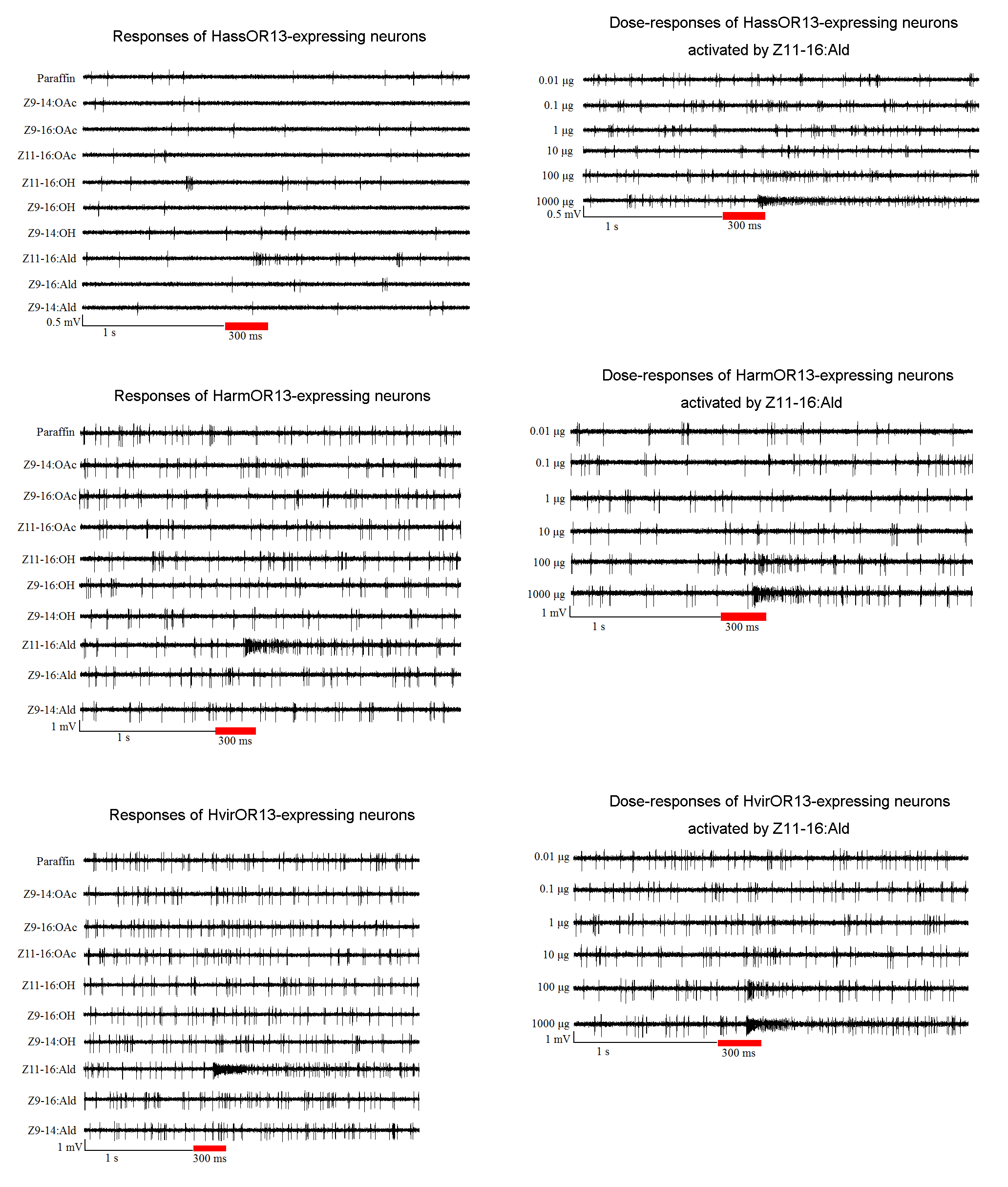

Supplement: FIGURE S2 — SSR traces from OR13-expressing neurons in at1 sensilla of Drosophila in response to pheromone compounds. [file Image_2.TIF]

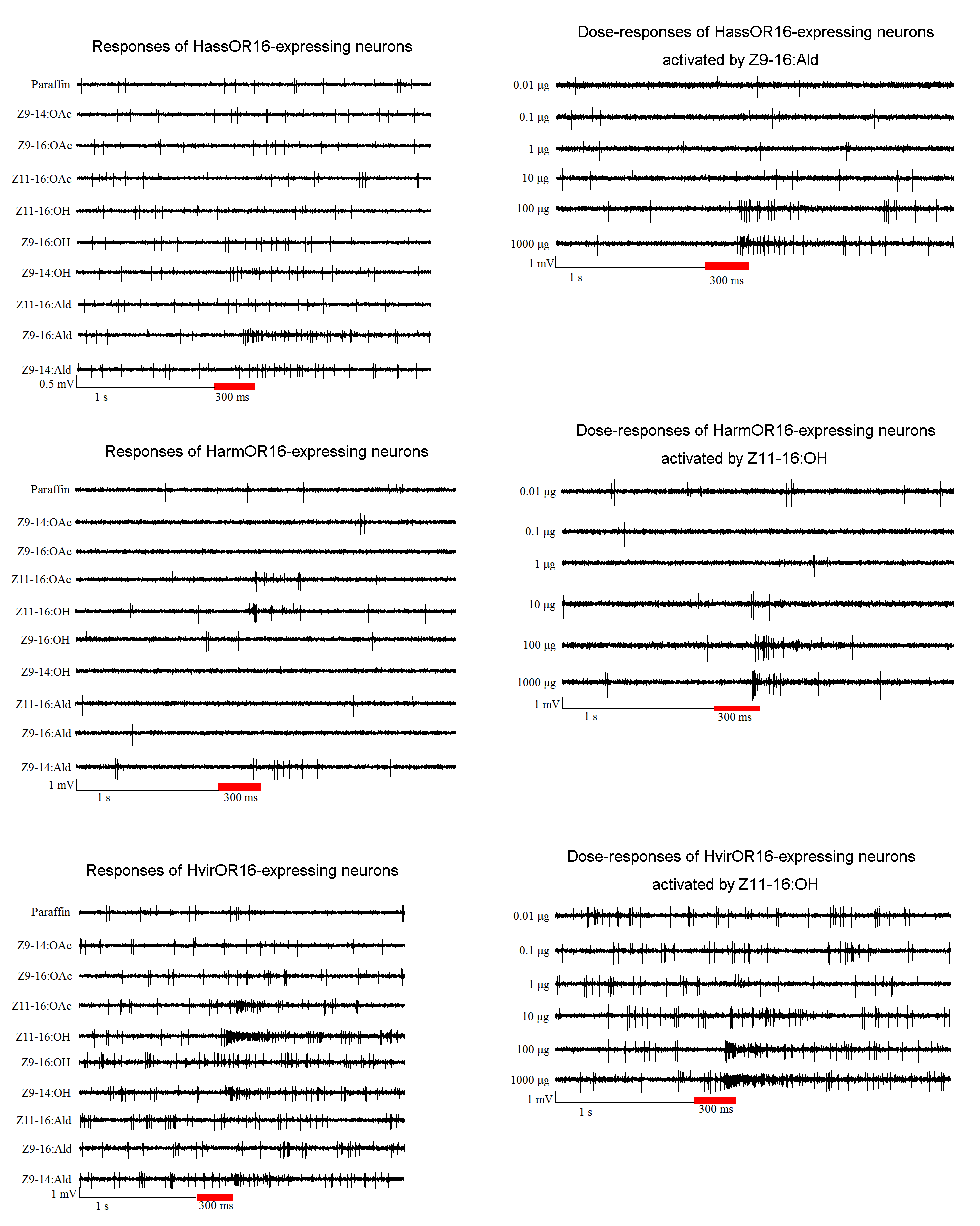

Supplement: FIGURE S3 — SSR traces from OR16-expressing neurons in at1 sensilla of Drosophila in response to pheromone compounds. [file Image_3.TIF]
