# Supplementary material for: Proceeding From in vivo Functions of Pheromone Receptors: Peripheral-Coding Perception of Pheromones From Three Closely Related Species, Helicoverpa armigera, H. assulta, and Heliothis virescens
Source: Front Physiol. 2018 Aug 30;9:1188. doi: 10.3389/fphys.2018.01188 (PMC6125646; doi:10.3389/fphys.2018.01188)
Supplement: TABLE S1 — Functional characterizations of PR genes in some Lepidopteran species. [file Table_1.docx]

Table S1. Functional characterizations of PR genes in some Lepidopteran species

| Expression system | | Species | Genes | References |
| --- | --- | --- | --- | --- |
| ***In vitro*** | *Xenopus* oocytes | *Helicoverpa armigera* | HarmOR6  HarmOR14b | Jiang et al., 2014 |
|  |  | *H. armigera* | HarmOR6  HarmOR11  HarmOR13  HarmOR14  HarmOR15  HarmOR16 | Liu et al., 2013b |
|  |  | *Helicoverpa assulta* | HassOR6  HassOR13  HassOR16 | Jiang et al., 2014 |
|  |  | *H. assulta* | HassOR13 | Wang et al., 2016 |
|  |  | *H. assulta* | HassOR6  HassOR13  HassOR16 | Chang et al., 2016 |
|  |  | *Spodoptera exigua* | SexiOR6  SexiOR11  SexiOR13  SexiOR16 | Liu et al., 2013a |
|  |  | *Bombyx mori* | BmOR1 | Nakagawa et al., 2012 |
|  |  | *B. mori* | BmOR1  BmOR3  BmOR4  BmOR5  BmOR6 | Nakagawa et al., 2005 |
|  |  | *B. mori* | BmOR1 | Sakurai et al., 2004 |
|  |  | *Heliothis virescens* | HvOR6  HvOR11  HvOR13  HvOR14  HvOR15  HvOR16 | Wang et al., 2011 |
|  |  | *Spodoptera litura* | SlituOR6  SlituOR11  SlituOR13  SlituOR16 | Zhang et al., 2015b |
|  | HEK293 cells | *B. mori* | BmOR1  BmOR3 | Grosse-Wilde et al., 2006 |
|  |  | *H. virescens* | HR13 | Grosse-Wilde et al., 2007 |
|  |  | *H. virescens* | HR13 | Pregitzer et al., 2012 |
| ***In vivo*** | *Or67d*^GAL4^ knock-in system | *Spodoptera littoralis* | SiltOR6  SlitOR13 | de Fouchier et al., 2015 |
|  |  | *S. littoralis* | SiltOR6 | Montagné et al., 2012 |
|  |  | *B. mori* | BmorOR1 | Syed et al., 2010 |
|  |  | *H. virescens* | HvOR13 | Vasquez et al., 2013 |
|  |  | *H. assulta* | HassOR13 | Wang et al., 2016 |
|  | Orco-GAL4/ UAS-OR lines | *S. littoralis* | SiltOR6 | Montagné et al., 2012 |
|  |  | *S. littoralis* | SiltOR6 | Ueira-Vieira et al., 2014 |
|  |  | *B. mori* | BmorOR1 | Ueira-Vieira et al., 2014 |
|  | “empty neuron” system | *B. mori* | BmorOR1 | Syed et al., 2006 |
