# Supplementary material for: Proceeding From in vivo Functions of Pheromone Receptors: Peripheral-Coding Perception of Pheromones From Three Closely Related Species, Helicoverpa armigera, H. assulta, and Heliothis virescens
Source: Front Physiol. 2018 Aug 30;9:1188. doi: 10.3389/fphys.2018.01188 (PMC6125646; doi:10.3389/fphys.2018.01188)
Supplement: TABLE S2 — Functional characterizations of PRs in three Heliothis/Helicoverpa species in vitro. [file Table_2.docx]

Table S2. Summary on *in vitro* functional characterizations of PRs in three Heliothis/Helicoverpa species in previous work

| Species | Functional identification | | | | | | |
| --- | --- | --- | --- | --- | --- | --- | --- |
|  | OR6 | OR11 | OR13 | OR14 | OR14b | OR15 | OR16 |
| *H. armigera* | F ^a,b^ | N ^a^ | F ^a^ | N ^a^ | F ^b, d^ | N ^a^ | F ^a^ |
| *H. assulta* | F ^b^ | N ^b^ | F ^b^ | * | N ^b,d^/ F ^e^ | — | F ^b^ |
| *H. virescens* | F ^c^ | N ^c^ | F ^c^ | F ^c^ | — | N ^c^ | F ^c^ |

“F” represents function; “N” represents no function; “—” represents lack of OR. “*” represents no test.

Refs: ^a^ (Liu et al., 2013); ^b^ (Jiang et al., 2014), ^c^ (Wang et al., 2010); ^d^ (Chang et al., 2016); ^e^ (Yang et al., 2017).
